# The need for strong clinical leaders – Transformational and transactional leadership as a framework for resident leadership training

**DOI:** 10.1371/journal.pone.0183019

**Published:** 2017-08-25

**Authors:** Barbara Saravo, Janine Netzel, Jan Kiesewetter

**Affiliations:** 1 Institut für Didaktik und Ausbildungsforschung in der Medizin, Klinikum der Ludwig-Maximilians-Universität München, Munich, Bavaria, Germany; 2 Center for Leadership and People Management, Ludwig-Maximilians-Universität München, Munich, Bavaria, Germany; University of Freiburg, GERMANY

## Abstract

**Background:**

For the purpose of providing excellent patient care, residents need to be strong, effective leaders. The lack of clinical leadership is alarming given the detrimental effects on patient safety. The objective of the study was to assess whether a leadership training addressing transactional and transformational leadership enhances leadership skills in residents.

**Methods:**

A volunteer sample of 57 residents from postgraduate year one to four was recruited across a range of medical specialties. The residents took part in an interventional controlled trial. The four-week IMPACT leadership training provided specific strategies for leadership in the clinical environment, addressing transactional (e.g. active control, contingent reward) and transformational leadership skills (e.g. appreciation, inspirational motivation).

Transactional and transformational leadership skill performance was rated (1) on the Performance Scale by an external evaluator blinded to the study design and (2) self-assessed transformational and transactional leadership skills. Both measures contained items of the Multifactor Leadership Questionnaire, with higher scores indicating greater leadership skills.

**Results:**

Both scores were significantly different between the IMPACT group and the control group. In the IMPACT group, the Performance Scale increased 15% in transactional leadership skill performance (2.10 to 2.86) (intervention effect, 0.76; 95% CI, 0.40 to 1.13; *p* < .001, eta^2^ = 0.31) and 14% in transformational leadership skill performance (2.26 to 2.94) (intervention effect, 0.68; 95% CI, 0.27 to 1.09; *p* < .001, eta^2^ = 0.22). The self-assessed transactional skills revealed a 4% increase (3.83 to 4.03) (intervention effect, 0.20; 95% CI, 0.08 to 0.33; *p* < .001, eta^2^ = 0.18) and a 6% increase in transformational leadership skills (3.54 to 3.86) (intervention effect, 0.31; 95% CI, 0.02 to 0.40; *p*< .001, eta^2^ = 0.53).

**Discussion and conclusions:**

These findings support the use of the transactional and transformational leadership framework for graduate leadership training. Future studies should incorporate time-latent post-tests, evaluating the stability of the behavioral performance increase.

## Introduction

There is currently a consensus that young physicians are in need of training in how to be effective leaders[1–5]. Medical residents take on various leadership responsibilities in their daily clinical work. For instance, they negotiate care plans, teach medical trainees, balance diverging perspectives in multiprofessional teams, while providing effective, safe delivery of care[5, 6]. Residents’ leadership skills are not facilitated enough [5]. This is surprising, given the body of evidence that highlights the association between effective leadership and the improvement of medical care in fields such as teamwork[7–9], communication[3] and patient safety[10, 11].

Current perspectives on clinical leadership are not precisely conceptualized. Existing concepts mainly reflect traditional understandings of the leader-follower relationship, focusing on individual behaviors and positional power[12]. As physicians in training usually do not hold formal authority and their leadership roles are not clearly defined[13], a distinct approach in framing the concept of leadership is needed[6]. Through a more precise concept of leadership, specific leadership behaviors could be identified and trained.

A vast number of leadership theories have been established in the organizational literature[14]. However, the medical community is facing the challenge of identifying appropriate concepts, and of adjusting them to the complex clinical environments in order to design target-oriented training programs. For graduate medical education, it has been recommended to base leadership training programs on established best practices[5, 15].

A few attempts in developing resident leadership training have been made. However, as has been found in a current review on leadership training in undergraduate medical education[15], most programs vary greatly in aligning the curricula with competencies. Also, existing studies rarely associate outcomes to the interventions[16, 17]. Leadership programs for residents mostly adhere to broad dimensions, such as confidence[18], communication skills[3], emotional intelligence[19], or postgraduate careers[20] and organizational leadership[21]. To date, there is no systematic, evidence-based knowledge about effective strategies to cultivate outstanding, strong leaders in residency.

In this article, we introduce the *Full Range Leadership Model* (FRLM)[22] into resident leadership training. It is the best empirically studied and most significant leadership framework in organizational literature and has been successfully implemented in several fields of application[23–27]. First efforts have been made to empirically test the model in medicine, however these have primarily addressed nursing leadership[28, 29] and hospital management[30–32].

According to the authors of the FRLM[33], a specific set of leadership components is necessary for effective leadership: a large portion of transformational leadership, higher levels of transactional leadership and a minimum of passive leadership. *Transformational leadership* refers to leaders with an appealing vision for their team who intellectually stimulate others in a way that is demanding and appreciative of the individual needs of the team members[14]. *Transactional leaders* exert influence on followers based on exchanging benefits for outstanding performance and response to their self-interests when they have achieved defined goals[34]. In contrast to transformational and transactional leadership, Bass[35] defines leaders who do not take charge of their leadership role as *passive leaders*. These three components of leadership are conceptualized as different levels of activity a leader can display, with passive leadership as the least active form of leadership[36]. For example, passive leaders avoid intervening when mistakes are made and do not execute managerial functions[37]; especially in high-stakes organizations like medical care, passive leadership can have harmful consequences[38]. On the contrary, transactional leaders reinforce their leadership by exercising active control when problems arise. Given the detrimental effects of passive leadership, we consider transactional and transformational leadership a crucial part of clinical leadership, where patient safety is among the highest of priorities. Transformational and transactional leadership are conceptualized as two distinct, yet interrelated components of leadership behavior[39]. Therefore, leadership programs should address both components alike.

We argue that, for ensuring high-quality delivery of care and for maximizing clinical productivity, physicians are expected to formulate clear expectations, set high standards and motivate team members to make strides to meet specified requirements. If, for example, followers get a feeling of involvement and are rewarded for making good efforts, they are more likely to be eager to achieve the goals that have been set. A clinical leader should both have the capacity to be transformational and transactional, but always be able to exert active control when needed.

Prior research has found positive effects of transformational and transactional leadership on several outcomes, such as enhanced satisfaction[40], the willingness of followers to generate extra effort[41], and increased performance[27].

While prior research showed that transformational, transactional and passive leadership are applicable in evaluating leadership styles in residents[42] and senior physicians[43], so far, no study has examined whether the model is suitable to guide resident leadership training and advance clinical leadership.

Gabel[13] particularly calls for training programs for informal leaders addressing transformational leadership. In our four-week IMPACT leadership training for residents, we explicitly tied those transformational and transactional leadership skills to the curriculum that are most relevant for everyday clinical practice. The training curriculum included the acquisition of key leadership knowledge, application of practical leadership skills, and simulation-based role-plays representing real performance situations of inpatient teams.

### Objectives

We hypothesize that over the course of the IMPACT training, (1) residents’ performance of transformational and transactional leadership skills as rated by an external evaluator will improve, (2) self-assessed transformational and transactional leadership skills will increase, and (3) residents’ knowledge on leadership will expand.

## Methods

### Sample

For organizational reasons we split the IMPACT training group into four cohorts, with each cohort consisting of 10–15 persons. Cohort one and two took part in the IMPACT leadership training program in February/March 2015 and cohort three and four in August/September 2015. All residents at our institution were eligible for enrollment in the study. Participation was voluntary and free of charge. For inclusion in the study, residents had to be 1) affiliated to one of the clinics of the university hospital, 2) in residency training for up to four years, and 3) willing to participate in all four consecutive training sessions. 57 residents were included in the study, representing a range of specialties: internal medicine, pediatrics, surgery, psychiatry, anesthesiology, neurology, radiology, gynecology, dermatology and ophthalmology. Exclusion criteria were 1) affiliation to an institution other than a university hospital, 2) in residency training for more than four years, 3) not committing to participation in all training sessions. According to these criteria, six applicants had to be excluded from study entry. Participants of the control group (*n* = 23) were recruited via email listings between cohort two and three, after all available positions for the IMPACT group had been assigned.

### Training procedure

The training design and procedure was based on and adapted from a leadership training for final year medical students[44, 45]. The IMPACT leadership training was conducted over four consecutive weeks, with two-and-a-half hour sessions once a week after clinical duties. To ensure instructional training efficiency, we relied on the same three instructors across all training cohorts. Instructors either came from a leadership training background (JN) or a medical education background (BS, JK).

We designed the training in four modules. Module one introduced leadership theory, focusing on transactional and transformational leadership and reflection on the residents’ leadership role within their clinical team. Module two tested the participants’ leadership behavior in one of four standardized five-minute scenarios in a simulation-based environment. The scenarios were carried out with professional actresses who were specially trained and functioned as nurses within the role-plays. Female actresses were chosen for role-plays representing daily practice in German hospitals where the majority of nurses is female[46]. The scenarios originated from a critical incident study[44], were randomly assigned to the residents and were recorded on video for later evaluation. Given the positive effects of feedback on training transfer[26, 47], we integrated a half-hour one-on-one feedback session between modules two and three; based on the recorded role-plays, physicians reflected their leadership performance together with their instructor. Module three comprised practicing communication techniques explicitly tied to transactional and transformational leadership. Module four tested the participants in another standardized scenario within the simulation-based environment. Actresses and scenarios were evenly distributed over module two and module four.

At intervals of four weeks, participants of the control group filled out two online questionnaires containing the same self-assessment scales as those of the IMPACT group and received a manuscript regarding clinical leadership. All participants of the control group received €40 in compensation after completion.

### Study design and setting

This study was a single-institution repeated measurement controlled trial at a large university hospital during the year 2015 involving several different clinics of various medical specialties.

The primary outcome was transactional and transformational leadership skill performance as assessed by an external evaluator, the secondary outcome self-evaluated transformational and transactional leadership skills, and leadership knowledge. In the training group (IMPACT group), the primary and secondary outcomes were tested in a pre-post design. Measurement took place before the training and four weeks after the first training session.

In the control group, only the secondary outcome was assessed pre-to-post-test within a four week interval, as residents of the control group did not receive any role-play intervention.

Ethical approval for the study was obtained from the ethical committee of the Ludwig-Maximilians-University (LMU) Munich. Prior to the training, all residents gave their written informed consent in study participation.

### Outcome measures

For the 12-item Performance Scale (measuring the primary outcome), we designed items that represented target behaviors for transactional and transformational clinical leadership. For instance, target behaviors for transactional leadership were tested by statements such as ‘The physician gave positive feedback for good performance’; transformational leadership was assessed by items like ‘The physician talked about the goals that have been set in an encouraging way.’. An external evaluator, who was blinded to the assignment and specially trained to assess (i.e. video coding), rated the primary outcome. The evaluator rated the recorded role-plays on a five-point Likert scale (range, 1 = strongly disagree to 5 = strongly agree). Examples for the ratings and corresponding leadership skills (i.e. target behaviors) are presented in Table 1. Rating quality was ensured by a ten percent inter-rating (*ICC* = 0.92).

**Table 1:** Example leadership skills of the performance scale by leadership component.

Leadership component	Leadership skill
**Transactional leadership**	The resident…
	• gave positive feedback for good efforts.
	• made clear what the nurse can expect when she performs well.
	• clarified who is responsible for defined tasks.
**Transformational leadership**	• treated the nurse respectfully.
	• encouraged the nurse to engage in overall goals of the clinical team.
	• formulated an appealing vision of what shall be achieved to improve patient care.

The 40-item Leadership Scale (assessing the secondary outcome) includes three subscales: transactional, transformational and passive leadership, containing items of the German version of the Multifactor Leadership Scale[48], a valid tool to evaluate the FRLM. Before and after four weeks of training, participants stated on a five-point Likert scale (range, 1 = not at all to 5 = frequently, if not always) the frequency with which they exerted a certain leadership behavior.

The nine-item multiple choice (measuring the secondary outcome) knowledge test was developed to examine leadership knowledge regarding transformational and transactional leadership. The test yields a composite mean score of 34.

A-priori, all scales were validated in a pilot study. Residents of different fields of application (e.g. anesthesia, surgery, radiology) were interviewed one-by-one after filling out the scales of the subjective measurement. Based on their ratings and comments, we revised the Leadership Scale as well as the multiple choice knowledge test thoroughly and made changes where necessary.

### Data collection

Prior to the training, participants received a random pseudonym as an identifier on data sheets. Residents participating in the IMPACT group filled out the Leadership Scale as well as the knowledge test on-site before and after the training, and were supervised by one of the trainers at any time. Within an interval of four weeks, participants of the control group filled out these two tests, as well. However, data of the control group were only obtained via online assessment. As participants of the control group filled out the tests at home or at their workplace, supervision could not be established. The Performance Scale was assessed by an independent evaluator after modules two and four. The dataset underlying our results is publicly available from the data repository Open Data LMU (DOI: https://doi.org/10.5282/ubm/data.109).

### Statistical analysis

A sample size calculation was performed and it was found that a sample size of 60 was needed to provide 80% power to detect medium to large effects. Data was entered into SPSS (version 23.0, SPSS Inc. Chicago, Illinois) for further analysis. We used repeated measures ANOVA to test for (1) the progression of externally rated leadership skills and self-assessment of the leadership scale as well as of the knowledge test, and (2) group between control and IMPACT group for the leadership knowledge test and self-assessment of the leadership-scale. All analyses were based on a 5% level of significance.

## Results

Of 57 residents, 50 (88%) completed the training (*m* = 29.98 years; *SD* = 2.60), with 25 (50%) female participants; 40 residents (70%) performed both role-plays. Reasons for missing sessions included clinical emergencies, unexpected changes in rotation schedules, clinical examination of incoming refugees at the central station, or illness. 23 residents participated in the control-group (*n* = 23; *m* = 29.13 years; *SD* = 2.53), with 18 (78%) female residents. All outcome variables showed sufficient reliability, with a pre-test Cronbach’s alpha of 0.55 (knowledge test), 0.64 (transactional leadership) and 0.85 (transformational leadership) and a post-test Cronbach’s alpha of 0.70 (transactional leadership), 0.81 (knowledge test) and 0.84 (transformational leadership).

### Performance scale

**Fig 1:**
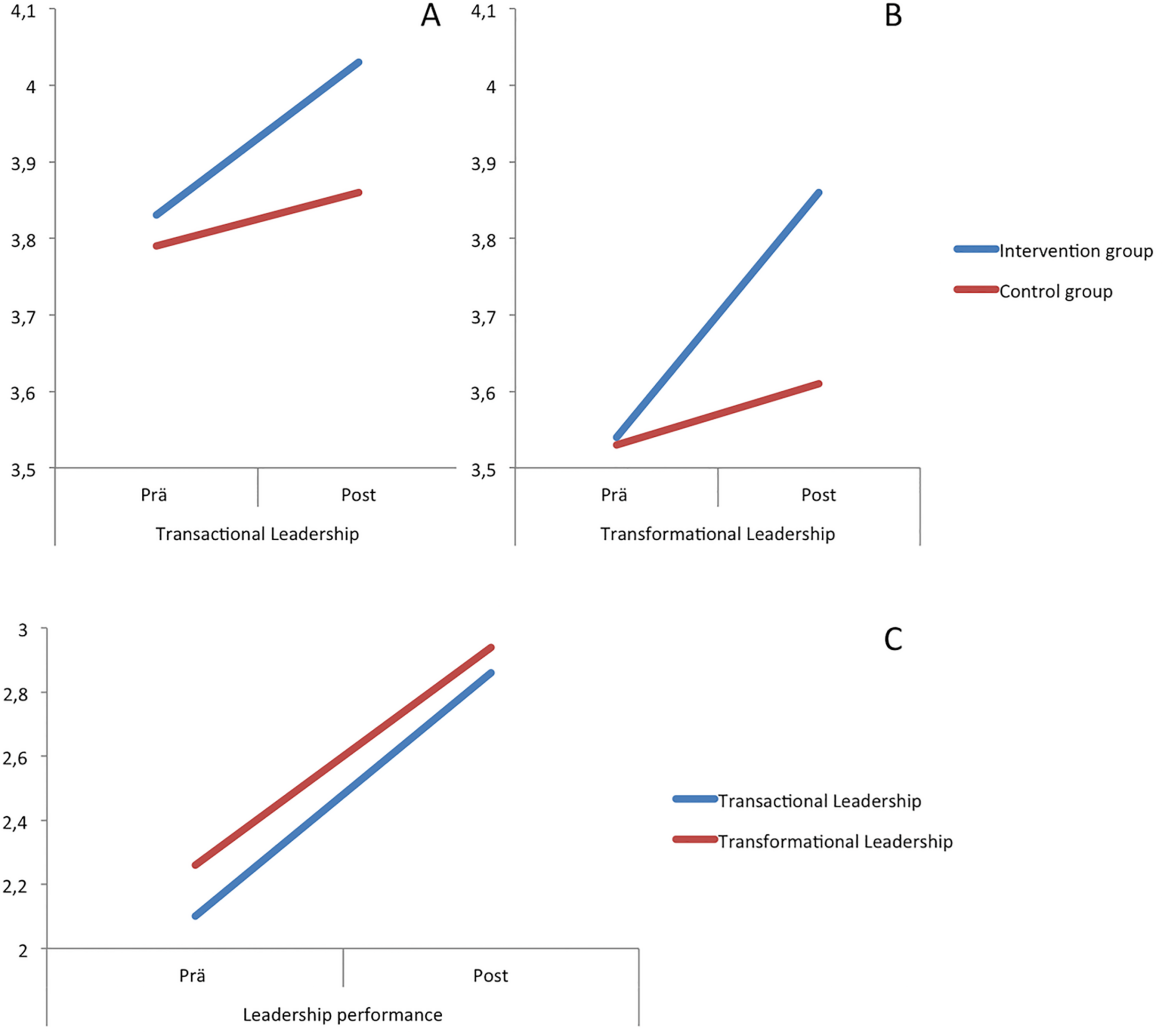
Overview of the effect for the self assessed leadership scale (graph A and B) and the performance scale (graph C).

As hypothesized, after four weeks of training, the Performance Scale increased 15% in transactional leadership skill performance (2.10 to 2.86) (intervention effect, 0.76; 95% CI, 0.40 to 1.13; *P*< .001, eta^2^ = 0.31) and 14% in transformational leadership skill performance (2.26 to 2.94) (intervention effect, 0.68; 95% CI, 0.27 to 1.09; *P*< .001, eta^2^ = 0.22). Table 2 presents pre- and post-test means, standard deviations and mean changes for leadership skill performance. Graph C illustrates this effect in Fig 1.

**Table 2:** Descriptive statistics and mean changes for leadership skill performance, divided into transactional and transformational leadership.

Performance Scale External Rater	*n*	*m*	*SD*	mean change (95% CI)
**Transactional leadership**^a^				
Pre-test to post-test	40			0.76 (0.40, 1.13)
Pre-test	40	2.10	0.75	
Post-test	40	2.86	0.99	
**Transformational leadership**^a^				
Pre-test to post-test	40			0.68 (0.27, 1.09)
Pre-test	40	2.26	0.88	
Post-test	40	2.94	1.13	

a Scale: 1 = strongly disagree, 2 = disagree, 3 = neutral, 4 = agree, 5 = strongly agree; *m* = mean; *SD* = standard deviaton; *n* = sample size; *CI* = confidence interval

### Leadership scale

Expectedly, we found a significant increase in self-assessed transactional and transformational leadership, as well as a significant interaction between point of measurement and group, indicating a substantial gain in both leadership components only for the IMPACT group (*F*(1,73) = 5.63, *P*< .02, eta^2^ = 0.07). The Leadership Scale revealed a 4% increase in self-assessed transactional skills (3.83 to 4.03) (intervention effect, 0.20; 95% CI, 0.08 to 0.33; *P*< .001, eta^2^ = 0.18) and a 6% increase in transformational leadership skills (3.54 to 3.86) (intervention effect, 0.31; 95% CI, 0.02 to 0.40; *P*< .001, eta^2^ = 0.53). The reported effects are considered to be excellent[49]. Mean scores for passive leadership were low at baseline, both in the IMPACT group (*m* = 1.87, *SD* = 0.55) and the control group (*m* = 1.94, *SD* = 0.51). Graph A and B illustrate this effect in Fig 1.

### Knowledge test

Two cases had to be eliminated from further analysis as data sets were not complete. Groups differed significantly in a-priori (*F*(1,69) = 17.17, *P*< .001, eta^2^ = 0.20) and post-test leadership knowledge (*F*(1,69) = 15.51, *P*< .001, eta^2^ = 0.19). Testing for group differences for the leadership knowledge test resulted in a significant effect for group (*F*(1,69) = 22.26, *P*< .001, eta^2^ = 0.25), yet there was no significant increase in leadership knowledge in any of the groups. In Table 3, pre- to post-test findings of transactional, transformational leadership, as well as leadership knowledge are summarized.

**Table 3:** Improvement of transactional and transformational leadership skills in the IMPACT group and changes in leadership knowledge, as compared to a control group.

	IMPACT Group			Control Group		
Scale	mean change (95% CI)	*m*	*SD*	mean change (95% CI)	*m*	*SD*
**Transactional leadership**^a^						
Pre-test to post-test^c^	0.20 (0.08, 0.33)			0.07 (0.10, 0.23)		
Pre-test		3.83	0.48		3.79	0.47
Post-test		4.03	0.45		3.86	0.53
**Transformational leadership**^a^						
Pre-test to post-test^c^	0.31 (.023, 0.40)			0.83 (0.08, 0.25)		
Pre-test		3.54	0.38		3.53	0.48
Post-test		3.86	0.31		3.61	0.40
**Knowledge test**^b^						
Pre-test to post-test^d^	0.6 (-1.17, 1.29)			-1.09 (-2.96, 0.77)		
Pre-test		26.48	2.67		22.95	4.32
Post-test		26.54	4.19		21.86	5.28

a Scale: 1 = not at all, 2 = once in a while, 3 = sometimes, 4 = fairly often, 5 = frequently.

b Scale: Multiple choice format, maximum achievable score = 34

c Significance level: *p* < .01

d Significance level: *p*>.30

m = Mean, *SD* = Standard Deviaton, *CI* = confidence interval.

### Correlations between the scales

In line with prior studies[25, 50], the two subscales of the leadership scale, transactional and transformational leadership, correlated significantly (*r* = .60; *P*< .05). Accordingly, passive leadership showed negative, significant correlations to transformational (*r* = -.49; *P*< .001) and transactional leadership (*r* = -.52; *P*< .001).

## Discussion

In this study, the four-week IMPACT leadership training was designed to increase transformational and transactional leadership skills in residents of various specialties. Transformational leadership refers to the leader’s ability to motivate team members to commit to a common goal. Transactional leadership involves the practice of exchanging benefits for excellent performance. Taking into account that a comprehensive set of leadership skills is necessary to meet the complex demands of daily clinical practice[5], we based our training on the Full Range Leadership Model[22].

Previous studies on leadership trainings have mainly been conducted in the organizational setting, primarily focusing on the transformational leadership component. The study by Abrell et al.[26] can be considered as one of the most comprehensive effort to train transformational leadership in a long-term study design. In their study, transformational leadership was assessed by subordinates and leadership performance was rated by leaders’ supervisors, showing a significant improvement over time[26]. Abrell et al.[26] incorporated feedback mechanisms into their curriculum as well as theoretical sessions giving an in-depth review of different transformational leadership styles, such as ‘individual consideration’ or ‘inspirational motivation’. To the best of our knowledge, there is no program training transformational and transactional leadership alike, neither in the organizational nor in the medical field of application.

Existing leadership programs in graduate medical education, such as the one by Awad et al.[3], focus on broader communication skills. Awad et al.[3] implemented a leadership training for surgical residents over the course of 6 months. They aimed at improving collaborative leadership through fostering a communication style that is regarded less commanding. Before and after completion of the training, residents assessed self-perceived alignment of the team, communication and integrity. The authors were able to demonstrate significant increases in these areas; however, training effects in terms of leadership performance such as improved team interactions have not been evaluated.

Our results go beyond prior research in different ways: First, unlike in the study by Abrell et al.[26], we not only tested for transformational leadership skills but also for transactional leadership skills in a pre-post design. Second, for the first time, the two leadership components were trained in a group of residents, extending external validity of the proposed leadership model to the medical education area of application. Overall, our results indicate that the FRLM is well suited for empirically testing leadership skills in residents of a wide spectrum. Third, we built upon first attempts to test the model in the medical context[42, 43] by providing a targeted, multimethod, structured training curriculum to improve resident leadership. Fourth, the different evaluation data modalities we applied (self-assessment, evaluation of performance) expand existing studies that have not evaluated the behavioral component of leadership.

We provided evidence that both distinct leadership components laid forth in the model are applicable for displaying significant increases in residents’ leadership performance. For example, at the end of the training, residents were able to show appreciation for good efforts (*transformational leadership skills*) and make clear who is responsible for specific tasks (*transactional leadership skills*). Interestingly, residents scored higher in self-assessed transactional leadership at baseline than in transformational leadership. They did change significantly in both leadership components, yet remained higher mean scores for transactional leadership also after training was completed. We believe this reflects the unique requirements of the clinical setting where fostering and sustaining patient safety is among the highest of priorities. In their everyday clinical practice, residents might feel more obliged to intervene and exert active control in order to prevent medical errors, thus exhibiting more transactional leadership behaviors.

Our results further suggest that four weeks of training seem to be a good starting point to effectively train leadership skills in residents across a wide range of specialties.

A control group did not increase in self-assessed leadership skills. It is remarkable that a substantial gain in both leadership components was demonstrated by video coding of simulations from an external evaluator perspective and by subjective data, as well. The increase in leadership skills from two different, independent perspectives supports the applicability of the leadership model for graduate medical education.

We controlled for a possible confounding effect of passive leadership at baseline, as this most ineffective leadership component is considered to attenuate the effect of transformational leadership on safety[38]. Consistent with previous studies[33, 42], mean scores for passive leadership were low in both groups.

### Limitations

Randomization and blinding between groups were not complete, as the participants were aware of which group they would be assigned to when applying; a selection bias can thus not be ruled out. However, this is somewhat ameliorated by the low and not differing scores between IMPACT group and control group as regards the leadership scale. For the knowledge test, there was no significant change in the two groups. Item difficulty for the pre-test measure might have been too high, resulting in low values of cronbach’s alpha at pre-test. In contrast, high values for cronbach’s alpha at post-test might be explained by increasing knowledge through the training. Apart from that, high standard deviations for mean scores for pre- and post-test might reflect a high range in leadership knowledge among the participants. A redesign of our knowledge test in terms of discriminatory power analysis might be beneficial in order to detect significant changes in residents’ leadership knowledge. In addition, both groups differed significantly in leadership knowledge at baseline and after the training. We traced this finding back to the different data acquisition modalities (on-site assessment versus unsupervised online assessment) and different levels of motivation. Unsupervised data collection also poses the problem of participants potentially consulting textbooks or the internet in order to gain better test results. For preventing potential confounding effects generated by data collection modalities, we suggest that future research should incorporate on-site data acquisition also for the control group. Passive leadership was only assessed prior to the training by the participants themselves. Since self-assessment is prone to self-serving biases, future studies should examine passive leadership also from an external perspective. As this study was a single-institution controlled trial, the degree of transferability could be enhanced by recruiting residents from different institutions. It must be noted, however, that our participants came from a wide range of specialties, and the proportion of female to male residents was balanced, indicating that the generalizability of our findings is not completely limited. Overall, our study would have benefited from a larger sample size, especially in the control group. However, the effect sizes for the group comparisons were extraordinarily high[49], raising questions as to the benefit of larger sample sizes.

## Conclusions

Our study is the first to establish and design a training format for the clinical setting based on the transactional and transformational leadership approach, going beyond previous research in a number of ways. First, we illustrated the feasibility of the proposed leadership framework for the clinical environment by providing evidence for support of this model. Second, we included a number of strong design elements, such as the use of a control group, and an outcomes assessment based on the performance rating by an external evaluator, as opposed to self-assessment. Third, in the following we provide best practice strategies for leadership programs specifically tailored for residents: As it is known that transactional and transformational leadership can have both a protective impact on patient safety[10, 11] and a positive effect on teamwork[7–9], medical institutions should establish resident leadership training drawn to the transactional and transformational framework. To ensure behavioral change, curricula should embed simulation-based practices, addressing transactional and transformational behaviors alike. Programs including one-on-one feedback can guide the way to individual high-quality leadership performance. Future studies should examine the implementation of the behavioral changes in daily clinical work, potentially incorporating leadership training as a starting point for mentoring programs within specialties[51]. We recommend building upon the target behaviors which we tied to transactional and transformational leadership skills in the performance assessment. We suggest to evaluate the stability of the increased leadership performance by conducting a time-latent post-test of the behavioral component in further studies. In order to expand upon the promising findings of our study, a research network for clinical leadership could prove beneficial for researchers to catalyze the design and evaluation of programs.
